# Diagnostic work-up of anemia and associated health outcomes in people with heart failure

**DOI:** 10.1186/s12916-025-04303-8

**Published:** 2025-08-12

**Authors:** Guobin Su, Ruowei Xiao, Dongze Ji, Kaiyu He, Anna Hallert, Gianluigi Savarese, Lars H. Lund, Yang Xu, Juan Jesus Carrero

**Affiliations:** 1 State Key Laboratory of Traditional Chinese Medicine Syndrome, Guangdong Provincial Key Laboratory of Chinese Medicine for Prevention and Treatment of Refractory Chronic Diseases, Big Data Research Center of Chinese Medicine, Department of Nephrology, Chinese Medicine Guangdong Laboratory, The Second Affiliated Hospital, the Second Clinical College of Guangzhou University of Chinese Medicine, Guangdong Provincial Hospital of Chinese Medicine, Guangzhou, China; 2 Department of Medical Epidemiology and Biostatistics, Karolinska Institutet, Stockholm, Sweden; 3 Department of Pharmacy Administration and Clinical Pharmacy, School of Pharmaceutical Sciences, Peking University Health Science Center, Beijing, China; 4 Särna VC, Dalarna, Sweden; 5 Department of Clinical Science and Education, Södersjukhuset, Karolinska Institutet, Stockholm, Sweden; 6 Department of Medicine, Section of Cardiology, Karolinska University Hospital, Stockholm, Karolinska Institutet Sweden; 7 Division of Nephrology, Department of Clinical Sciences, Danderyd Hospital, Danderyd, Sweden

**Keywords:** Heart failure, Anemia, Iron deficiency, Diagnostics, Cancer, Clinical outcomes

## Abstract

**Background:**

Anemia is common in patients with heart failure (HF). Although iron testing is recommended, it is uncertain that solely emphasizing iron testing could result in lesser attention to other causes, like bleeding or cancer. This study aimed to evaluate the diagnostic work-up of incident anemia in patients with HF in routine care and associated health outcomes.

**Methods:**

Observational study of 8932 non-anemic adults with HF in Stockholm, Sweden, was quantified for incidence of anemia, diagnostic work-up (recognition, laboratory/invasive testing) and treatment across severity of anemia and setting of care. Time-varying Cox regression explored associations between developing anemia and rate of major adverse cardiovascular events (MACE), HF hospitalization, cancer, and death.

**Results:**

During median 2.7 years, 34% of patients developed incident anemia, and 13% developed severe anemia. Within 6 months from incident anemia, ferritin and transferrin saturation were tested in 44% overall and 65% of severe cases. Testing of liver enzymes, creatinine, and C-reactive protein was, however, done in > 90% of cases. Colonoscopy, esophagogastroduodenoscopy, urinalysis, and cystoscopy were performed in 2–10% of cases. Few patients were recognized with an ICD code diagnosis of anemia (16%). Treatments were infrequent: oral iron (10%), intravenous iron (16%), blood transfusions (6%), and erythropoietin-stimulating agents (< 1%). More anemia cases received treatment in cardiology care (43%) versus primary care (29%). New-onset anemia was associated with risk of MACE (adjusted HR 2.13, 95% CI 1.85–2.44), HF hospitalization (4.85, 4.30–5.48), cancer (3.41, 3.09–3.77), and death (2.04, 1.82–2.29).

**Conclusions:**

One in three patients with HF experienced anemia, which was associated with adverse health outcomes. Testing for iron stores and invasive work-up was suboptimal. A large proportion of anemia events remained under-recognized and untreated, a pattern of care that warrants correction.

**Supplementary Information:**

The online version contains supplementary material available at 10.1186/s12916-025-04303-8.

## Background

Anemia is common in patients with heart failure (HF). In cross-sectional analyses, approximately 50% of patients with HF had prevalent anemia [1, 2]. Iron deficiency, a cause of anemia, is also common, ranging in cross-sectional studies from 35 to 49% [2, 3]. The presence of anemia and iron deficiency in HF has, independently of one another, been associated with lower life quality [4], increased rates of hospitalizations and death [5, 6]. Since 2016, the European Society of Cardiology (ESC) guidelines on HF recommend that physicians screen, diagnose, and treat iron deficiency, emphasizing testing for ferritin and transferrin saturation (TSAT) to identify functional iron deficiency [7]. Concerns have been raised that in HF, diagnosing and treating functional iron deficiency could detract attention from diagnosing serious underlying causes, such as cancer [8]. But it is also possible that expanded treatment indication in HF has increased attention to and diagnostic work-up in anemia. We speculate that solely emphasizing iron testing could result in lesser attention to other causes, like bleeding or cancer. Further, patients are seen by many specialists, including cardiologists and primary care physicians, who may be exposed to, familiar with, and follow different guidelines.

The concordance between guideline advice and anemia diagnostic work-up of patients with HF managed in routine care has not been sufficiently investigated. Available studies describe practices in the early 2012’s before guideline recommendations were issued [5]. Subsequent studies have focused on iron testing and iron deficiency management in patients with HF and *prevalent* anemia [2, 3, 5, 9–13]. Such approaches are limited by their cross-sectional nature, which can only evaluate completed actions across survivors or patients persistent to therapies. Other identified limitations include single-center studies [9], or clinical trial [10] or registry cohorts [2, 3, 5, 11, 12], which may have limited generalizability unlikely to reflect the heterogeneity of routine clinical practice. No studies have explored the incidence of anemia in HF patients, which more adequately allows for evaluating the quality of care processes and work-up to address this de novo complication.

We therefore performed an observational cohort study in the population with HF in Stockholm, Sweden. We assessed (i) the incidence of anemia, and which populations are at high risk; (ii) diagnostic work-up and treatment of anemia overall and across settings of care; and (iii) associations of developing anemia with adverse health outcomes.

## Methods

### Data source

We used data from the Stockholm Creatinine Measurements (SCREAM) project, which contains healthcare utilization data from all residents of the region of Stockholm, Sweden [13]. A single healthcare provider in the Stockholm region provides universal and tax-funded healthcare to 20–25% of the population of Sweden. SCREAM contains complete information on demographics, healthcare utilization, laboratory tests, dispensed drugs, diagnoses (captured by International Classification of Diseases 10th Revision (ICD-10) codes from primary care, specialist care and inpatient care records) and vital status [13].

### Study population

We included adults aged ≥ 18 years residing and accessing health care in Stockholm during 2016 to 2021 (last date available currently in SCREAM), with a diagnosis of HF and available subsequent hemoglobin tests. We excluded hemoglobin tests taken during an inpatient stay, or hemoglobin tests performed within 30 days after a bleeding event or a transfusion code and hemoglobin tests within 30 days from a hospitalization discharge, which could relate to the monitoring and/or resolution of an event. After these exclusions, we selected the date of the first hemoglobin test per patient as the index date for our study and the timepoint where baseline covariates were assessed, and follow-up began. At this point we further excluded patients who had conditions affecting the interpretation of hemoglobin values or of potential incident anemia-related outcomes, including recent pregnancy (within 2 years prior), ongoing or recent history of cancer (a clinical diagnosis of cancer in the previous 3 years, excluding diagnoses of melanomas), a hematologic disease, and chronic infections (e.g., hepatitis, tuberculosis, and human immunodeficiency virus) (Additional file 1: Table S1).

Once the study population had been defined, we identified and excluded individuals with anemia at the index date (i.e. prevalent anemia cases, defined by having a low hemoglobin value according to the World Health Organization (WHO) definition: < 12 g/dL for females or < 13 g/dL for males), or having received an anemia diagnosis (ICD-10 codes D50–D64) in the year prior to the index date or having received anemia treatment (erythropoietin stimulating agents (ESA) or iron) in the year prior to the index date (Additional file 1: Table S1). The resulting cohort was then a cohort of prevalent HF cases free from (recent) anemia and with a baseline hemoglobin value.

### Incidence of anemia and processes of care

This study comprised two analyses. The first analysis aimed to quantify the population with HF developing anemia and the processes of care around that anemia event. The primary outcome was thus the first-detected anemia event in each patient, defined as a new onset of low hemoglobin measurement (< 12 g/dL for females or < 13 g/dL for males) followed by a diagnosis of anemia, or initiation of anemia treatment (ESA or iron) within 3 months, or a subsequent hemoglobin measurement with similar magnitude between 3 and 6 months apart (i.e., anemia sustained for at least 3 months) (Additional file 1: Table S2). The event date was the date of the first low-detected hemoglobin. Anemia events were categorized by their severity: severe anemia was defined as hemoglobin < 10 g/dL regardless of sex followed by a diagnosis of anemia, treatment initiation, or a second hemoglobin measurement < 10 g/dL; mild/moderate anemia was defined as a hemoglobin measurement < 12 g/dL for females or < 13 g/dL for males but ≥ 10 g/dL, followed by a diagnosis of anemia, treatment initiation, or a second hemoglobin measurement of similar magnitude. Outcomes based on anemia severity were not mutually exclusive, and a given patient may have developed more than one anemia event of varying severity during follow-up.

The clinical work-up of anemia was evaluated in terms of (i) *anemia recognition*, defined by the establishment of a clinical diagnosis of anemia within 6 months from the anemia event; (ii) *testing for iron stores*, defined by the presence of at least one laboratory test of ferritin or TSAT within 6 months from the anemia event; (iii) *other laboratory testing*, including liver enzymes (alanine aminotransferase (ALT) and aspartate aminotransferase (AST), kidney function (serum/plasma creatinine) and inflammation (C-reactive protein (CRP)); (iv) *procedures for ruling out bleeding or cancer*, including colonoscopy, urinalysis, cystoscopy, and esophagogastroduodenoscopy; (v) *initiation of treatments*, including recorded blood transfusions, infusions of intravenous iron, and filled prescriptions of oral iron or ESAs within 6 months from the anemia event (definitions detailed in Additional file 1: Table S3).

### Clinical outcomes associated with incident anemia

The second analysis aimed to investigate the association between developing anemia and the subsequent risk of all-cause death, major adverse cardiovascular events (MACE) and hospitalization for HF, and new-onset cancer. Here, anemia was considered a time-varying exposure. Deaths were ascertained by linkage with the Swedish population register, which has no losses to follow up [14]. MACE was defined as the composite of stroke, myocardial infarction, and cardiovascular death. Cancer was defined as any new diagnosis falling under the International Classification of Diseases 10th Revision (ICD-10) C category (Additional file 1: Table S2).

### Study covariates and stratifiers

Study covariates were derived at index date and updated again at the time of anemia occurrence. Covariates were selected on the basis of biological plausibility and included demographic information (age, sex, highest level of education attained and calendar year); HF type, categorized as HF with preserved ejection fraction (HFpEF:EF ≥ 50%) or reduced ejection fraction (HFrEF: EF < 50%) by using a prediction model derived in a Swedish cohort and validated in a Dutch cohort [15] (Additional file 1: Method S1); history of comorbidities (diabetes mellitus, hypertension, ischemic heart disease, cerebrovascular disease (CVD), peripheral vascular disease, atrial fibrillation, valve disease, chronic obstructive pulmonary disease, rheumatoid diseases, dementia, liver disease, peptic ulcer disease and melanoma); laboratory tests (hemoglobin and estimated glomerular filtration rate (eGFR) [16]; and use of implantable cardioverter defibrillator or cardiac resynchronization therapy, use of CVD-related medications (renin–angiotensin system (RAS) inhibitors (angiotensin-converting enzyme (ACE) inhibitors/angiotensin II receptor blockers (ARBs)), beta-blockers, calcium channel blockers, loop diuretics, mineralocorticoid receptor antagonists (MRA), digoxin, statins, immunosuppressants, platelet aggregation inhibitors, anticoagulants except heparin, non-steroidal anti-inflammatory drugs, and other blood pressure medications. Detailed definitions of these covariates are presented in Additional file 1: Table S4.

We stratified our analyses by the presence/absence of iron deficiency and the type of care identifying the event (setting). Iron deficiency was defined as ferritin < 100 µg/L, or ferritin between 100 µg/L and 299 µg/L if TSAT was < 20% [17]. We considered that anemia was detected and managed in primary care if the hemoglobin test that defined the event was ordered by a primary care unit and there were no records of a cardiology department visit within 6 months; we considered that anemia was detected and managed in cardiology care if the hemoglobin test that defined the event was ordered by a cardiology department or if there was a recorded visit in a cardiology department within 6 months. For cases not fitting these definitions, we considered that anemia was detected and managed in other sources of care.

### Statistical analysis

Continuous variables with normal distribution are presented as mean and standard deviation (SD), whereas those with non-normal distribution are expressed as median and interquartile range (IQR). Categorical variables are presented as counts and proportions (%).

#### Incidence of anemia and baseline predictors

We first calculated anemia incidence rates by dividing the number of events by the person-time, following patients until the first anemia event detected. Then, we assessed time to anemia event, identifying baseline predictors (all those listed in Additional file 1: Table S5), through multivariable Cox regression models, reported as hazard ratios (HRs) and 95% confidence intervals (CIs). For these analyses, patients were followed until the occurrence of anemia, emigration from Stockholm, death, or end of follow-up, whichever occurred first. Continuous variables were standardized as per SD increase, and the relative importance of each predictor was assessed by the estimated explained variance of the outcomes (*R*^2^) and the proportion of overall explainable log-likelihood (*Χ*^2^) attributable to each predictor in the analysis of variance.

#### Clinical work-up of anemia

We described the clinical reactions upon anemia occurrence overall, stratified by anemia severity, by the absence/presence of iron deficiency, and by setting of management. Furthermore, we modelled these processes of care across calendar years to evaluate time trends since the instauration of the ESC guidelines in 2016. In addition, we reevaluated the clinical work-up of anemia after excluding patients who died within the first 6 to 12 months after incident anemia to account for the possibility that some of these patients may have been under palliative care, thereby limiting further diagnostic investigations and anemia treatment.

#### Adverse outcomes following incident anemia

Finally, we estimated the associations between developing anemia and subsequent outcomes through multivariable-adjusted time-dependent Cox proportional hazards regression. Patients were followed from the index date until the occurrence of adverse outcomes, emigration from Stockholm, or the end of follow-up, whichever occurred first.

To evaluate the consistency of our findings, we performed subgroup analyses by sex, HF type, and diabetes status. To evaluate the robustness of our findings, we performed various sensitivity analyses. First, we reanalyzed associations with all-cause death, MACE, hospitalized HF, and cancer after excluding events within the first 180 days after incident anemia to assess the impact of reverse causation bias (e.g., that anemia may have been identified during the workout and/or clinical investigations following another complication). Second, we reevaluated the associations between developing anemia and subsequent risk of MACE, hospitalized HF, and cancer, considering all-cause death as a competing risk.

#### Missing data approaches

There were no missing data for covariates except for educational level and baseline eGFR, with a missing rate of 3.4% and 1.9%, respectively (Additional file 1: Table S4). Multiple imputation by chained equations using classification and regression trees was employed to impute complete data sets. The imputation model included the exposure variable, all covariates, the event indicator for the outcome, and the Nelson-Aalen estimate of the baseline cumulative hazard.

Statistical analyses were performed using R Version 4.2.1 (R Foundation for Statistical Computing). Two-sided *P* < 0.05 was considered statistically significant.

## Results

### Baseline patient characteristics

There were 42,530 adults living with HF in Stockholm during 2016–2021. After applying inclusion and exclusion criteria, a total of 14,681 cases were evaluated at the time of first-identified testing for hemoglobin. At that point, 5621 (38.3%) patients were considered to have prevalent anemia and were further excluded, leaving 8932 adults with HF and free from anemia for inclusion in our study (Additional file 1: Fig. S1).

Their age was 78 ± 11 years. They were predominantly female (52.2%) and had a mean hemoglobin of 14.11 ± 1.21 g/dL. Half of them had HFrEF (51.8%), and 40.8% had eGFR < 60 mL/min/1.73 m^2^. The most common comorbidities were hypertension (88.9%), atrial fibrillation (52.8%), and ischemic heart disease (44.8%). The most common treatments were beta blockers (72.5%), ACE inhibitors/ARBs (71.1%), and loop diuretics (47.3%) (Table 1).

**Table 1:** Baseline characteristics of patients with heart failure

	**Overall**	**At time of first detected anemia**	**At time of first-detected mild/moderate anemia**	**At time of first-detected severe anemia**
Number of patients, *n *(%)	8932 (100)	3049 (34.1)	2970 (33.3)	1167 (13.1)
Age, mean (SD)	78 (11)	80 (10)	80 (10)	79 (10)
Age category, *n* (%)				
< 65 years	1044 (11.7)	225 (7.4)	219 (7.4)	93 (8.0)
≥ 65 to 79 years	3409 (38.2)	1132 (37.1)	1106 (37.2)	440 (37.7)
≥ 80 years	4479 (50.1)	1692 (55.5)	1645 (55.4)	634 (54.3)
Sex, female, *n* (%)	4666 (52.2)	1477 (48.4)	1426 (48.0)	667 (57.2)
Education level, *n* (%)				
Compulsory school	2827 (31.7)	990 (32.5)	967 (32.6)	396 (33.9)
Secondary school	3595 (40.2)	1229 (40.3)	1191 (40.1)	466 (39.9)
University	2208 (24.7)	723 (23.7)	706 (23.8)	262 (22.5)
Missing	302 (3.4)	107 (3.5)	106 (3.6)	43 (3.7)
Type of heart failure^a^, *n* (%)				
HFrEF	4631 (51.8)	1599 (52.4)	1568 (52.8)	576 (49.4)
HFpEF	4301 (48.2)	1450 (47.6)	1402 (47.2)	591 (50.6)
Hemoglobin at baseline, g/dL, mean (SD)	14.11 (1.21)	11.62 (1.09)	11.79 (0.77)	9.19 (0.94)
eGFR categories, median (IQR)	64.7 (49.7, 79.8)	57.9 (42.9, 75.3)	57.9 (42.7, 75.3)	53.82 (40.1, 73.0)
≥ 60 mL/min/1.73 m^2^, *n* (%)	5118 (57.3)	1355 (44.4)	1320 (44.4)	449 (38.5)
30–59 mL/min/1.73 m^2^, *n* (%)	3350 (37.5)	1309 (42.9)	1284 (43.2)	524 (44.9)
< 30 mL/min/1.73 m^2^, *n* (%)	291 (3.3)	226 (7.4)	219 (7.4)	122 (10.5)
Missing, *n* (%)	173 (1.9)	159 (5.2)	147 (4.9)	72 (6.2)
History of comorbidities, *n (%)*				
Diabetes	2736 (30.6)	1133 (37.2)	1112 (37.4)	457 (39.2)
Hypertension	7942 (88.9)	2796 (91.7)	2723 (91.7)	1085 (93.0)
Ischemic heart disease	4005 (44.8)	1510 (49.5)	1477 (49.7)	567 (48.6)
Cerebrovascular disease (includes stroke)	2100 (23.5)	825 (27.1)	804 (27.1)	312 (26.7)
Peripheral vascular disease	1311 (14.7)	622 (20.4)	613 (20.6)	273 (23.4)
Atrial fibrillation	4714 (52.8)	1864 (61.1)	1824 (61.4)	754 (64.6)
Valve diseases	892 (10.0)	436 (14.3)	428 (14.4)	200 (17.1)
Chronic obstructive pulmonary disease	2758 (30.9)	1118 (36.7)	1094 (36.8)	459 (39.3)
Rheumatoid diseases	2378 (26.6)	1066 (35.0)	1046 (35.2)	453 (38.8)
Dementia	700 (7.8)	285 (9.3)	274 (9.2)	127 (10.9)
Liver disease	235 (2.6)	92 (3.0)	87 (2.9)	57 (4.9)
Peptic ulcer disease	18 (0.2)	11 (0.4)	11 (0.4)	4 (0.3)
Melanoma	562 (6.3)	250 (8.2)	243 (8.2)	90 (7.7)
Ongoing treatments, *n (%)*				
Device therapies^b^	1431 (16.0)	636 (20.9)	621 (20.9)	246 (21.1)
ACE inhibitors/ARBs	6350 (71.1)	2209 (72.4)	2156 (72.6)	822 (70.4)
Mineralocorticoid receptor antagonists	1792 (20.1)	732 (24.0)	712 (24.0)	265 (22.7)
Loop diuretics	4226 (47.3)	1729 (56.7)	1687 (56.8)	712 (61.0)
Beta blockers	6478 (72.5)	2273 (74.5)	2218 (74.7)	886 (75.9)
Calcium channel blockers	2329 (26.1)	769 (25.2)	749 (25.2)	307 (26.3)
Digoxin	1043 (11.7)	338 (11.1)	323 (10.9)	154 (13.2)
Statins	3743 (41.9)	1363 (44.7)	1332 (44.8)	498 (42.7)
Platelet aggregation inhibitors	2981 (33.4)	952 (31.2)	931 (31.3)	353 (30.2)
Anticoagulants except heparin	3756 (42.1)	1542 (50.6)	1506 (50.7)	633 (54.2)
Non-steroidal anti-inflammatory drugs	629 (7.0)	223 (7.3)	215 (7.2)	79 (6.8)
Other blood pressure medication	105 (1.2)	49 (1.6)	47 (1.6)	23 (2.0)

Abbreviations: *ACE*, angiotensin-converting enzyme; *ARB*, angiotensin II receptor blocker; *eGFR*, estimated glomerular filtration rate; *HFpEF*, heart failure with preserved ejection fraction; *HFrEF*, heart failure with reduced ejection fraction; *IQR*, interquartile range; *SD*, standard deviation

Note: ^a^ Type of heart failure was predicted by the recent Swedish-based algorithm using eGFR levels after multiple imputation. ^b^ Device therapy includes: implantable cardioverter defibrillator or cardiac resynchronization therapy

### Incidence of anemia

During a median follow-up of 2.7 (IQR 1.1–4.9) years, 3049 (34.1%) patients developed incident anemia and 1167 (13.1%) developed severe anemia (Additional file 1: Table S6). Most anemia cases (58.4%) were identified by persistently low hemoglobin values, followed by anemia diagnoses (25.0%) or initiation of anemia treatments (16.6%). Multivariable Cox regression showed that older age, male sex, use of loop diuretics, and history of diabetes mellitus were the four baseline conditions mostly contributing to the prediction of incident anemia (Additional file 1: Fig. S2).

### Clinical work-up of anemia

Among 3049 anemia cases, only 15.7% received an ICD code diagnosis. The proportion of diagnoses issued was higher amongst patients with severe anemias (49.5%) compared to mild/moderate anemias (14.5%) (Table 2).

**Table 2:** Clinical work-up of anemia in patients with heart failure, overall and by anemia severity

	**Any anemia**	**Mild/moderate anemia**	**Severe anemia**
No. of anemia cases	3049	2970	1167
Received an anemia diagnosis,* n (%)*	480 (15.7)	431 (14.5)	578 (49.5)
Testing of iron stores, *n (%)*	1365 (44.8)	1312 (44.2)	762 (65.3)
Ferritin only	624 (20.5)	619 (20.8)	246 (21.1)
TSAT only	72 (2.4)	68 (2.3)	50 (4.3)
Ferritin and TSAT	669 (21.9)	625 (21)	466 (39.9)
Other recommend laboratoty testing, *n (%)*			
Liver enzymes (ALT or AST)	2522 (82.7)	2456 (82.7)	1009 (86.5)
Serum/plasma creatinine	3043 (99.8)	2965 (99.8)	1165 (99.8)
C-reactive protein	2789 (91.5)	2714 (91.4)	1118 (95.8)
Other recommend procedures, *n (%)*	542 (17.8)	518 (17.4)	402 (34.4)
Colonoscopy	130 (4.3)	121 (4.1)	120 (10.3)
Urinalysis	61 (2.0)	60 (2.0)	44 (3.8)
Cystoscopy	164 (5.4)	162 (5.5)	74 (6.3)
Esophagogastroduodenoscopy	313 (10.3)	286 (9.6)	307 (26.3)
Treatment patterns, *n (%)*			
Iron, oral	319(10.5)	307(10.3)	202(17.3)
Iron, intravenous	491(16.1)	488(16.4)	160(13.7)
Iron, oral + intravenous	33(1.1)	33(1.1)	48(4.1)
Blood transfusion	184 (6.0)	160 (5.4)	209 (17.9)
ESA	2 (0.1)	2 (0.1)	7 (0.6)
Combination treatment	160 (5.2)	130 (4.4)	268 (23.0)
Iron + blood transfusion	150 (4.9)	120 (4.0)	232 (19.9)
Iron + ESA	5 (0.2)	5 (0.2)	21 (1.8)
Blood transfusion + ESA	3 (0.1)	3 (0.1)	8 (0.7)
Iron + blood transfusion + ESA	2 (0.1)	2 (0.1)	7 (0.6)
Total population receiving any treatment	1189 (39.0)	1120 (37.7)	894 (76.6)

Abbreviations: *ALT*, alanine aminotransferase; *AST*, aspartate aminotransferase; *ESA*, erythropoietin-stimulating agent; *TSAT*, transferrin saturation

Testing for iron stores occurred in 44.8% of anemia cases. In the majority of cases, both TSAT and ferritin were tested concomitantly, followed by ferritin alone. The proportion of iron testing was higher amongst severe anemia cases (65.3%) compared with mild/moderate anemias (44.2%). Testing rates for other recommended biomarkers were much higher, with > 80% of cases undergoing testing for liver enzymes and > 90% of cases undergoing testing for CRP or creatinine. Few cases underwent procedures for detection of bleeding or cancer, but these tests were more common amongst severe anemias.

Overall, only 39.0% of anemia cases received treatment, primarily intravenous iron (16.1%), oral iron (10.5%), followed by blood transfusions (6.0%) and ESA (< 1%). The proportion of patients receiving treatment was higher amongst severe anemia cases (76.6%) compared to those with mild/moderate anemia (37.7%).

**Fig. 1:**
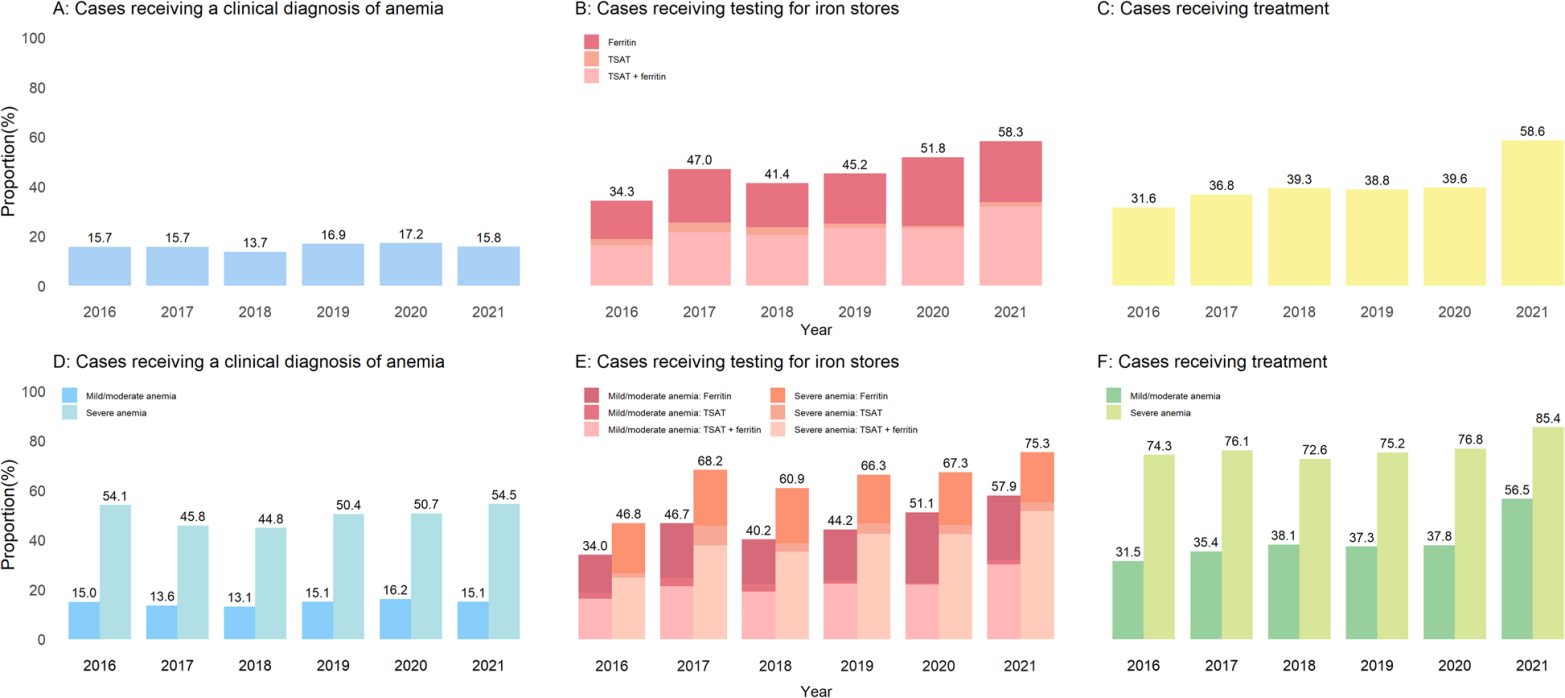
Time trends in clinical work-up of incident anemia amongst patients with heart failure. Abbreviations: TAST, transferrin saturation

During the period 2016–2021, there was little variation in the proportion of anemia events recognized with a diagnosis (Fig. 1). However, rates of ferritin or TSAT testing gradually increased, particularly the concomitant testing of both TSAT and ferritin. Treatment rates were rather stable, with approximately 38% of cases being treated every year, except for a peak in treatment rates in 2021.

#### Subgroup analyses

Compared with patients without iron testing, those receiving iron testing had a higher proportion of anemia diagnoses issued (8.9% vs. 24.2%) and more often received treatments (31.1% vs. 48.7%). Of patients receiving iron testing, 64.9% were identified as iron deficient. Iron deficient patients more often received iron therapy (48.8% of patients received iron alone or in combination with other treatments) than non-iron deficient patients (31.8%), but still a considerable proportion of cases remained untreated (Additional file 1: Table S7).

**Fig. 2:**
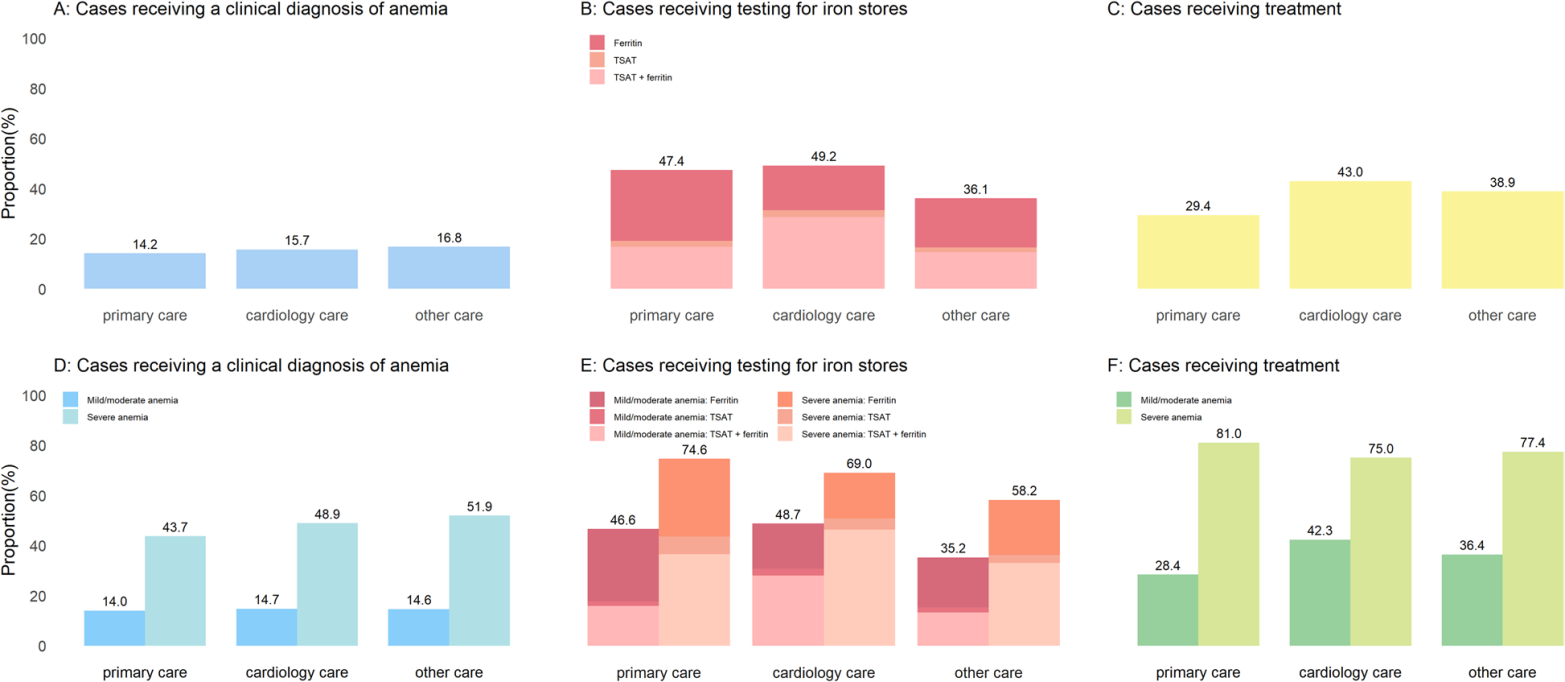
Clinical work-up of incident anemia amongst patients with heart failure by settings of management. Abbreviations: TAST, transferrin saturation

Across different settings of management, the proportion of cases recognized with a clinical diagnosis was similar. Patients managed in cardiology care (49.2%) and in primary care (47.3%) had infrequent testing for ferritin or TSAT. Other work-up indicators did not vary much across settings of management. More anemia cases received treatment in cardiology care (43.0%) versus those managed in primary care (29.4%) or other sources of care (38.9%) (Additional file 1: Table S8, Fig. 2).

The proportions of clinical work-up were similar after excluding patients who died within the first 6 to 12 months of incident anemia (Additional file 1: Tables S9).

### Adverse outcomes associated with incident anemia

During a median follow-up of 2.5 (IQR 1.0–4.5) years from incident anemia, 3452 (38.6%) patients died, 2436 patients (27.3%) developed MACE, and 3177 patients (35.6%) experienced a new HF hospitalization. After multivariable adjustment, developing anemia was associated with a twofold (HR 2.04, 95% CI 1.82–2.29) higher risk of all-cause death, a twofold (HR 2.13, 95% CI 1.85–2.44) higher risk of MACE, a close to fivefold (HR 4.85, 95% CI 4.30–5.48) higher risk of HF hospitalization, and a threefold risk of cancer (HR 3.41, 95% CI 3.09–3.77).

After excluding early events, the magnitude of the risk of all the adverse clinical outcomes decreased but remained statistically significant (Table 3). The magnitude of the associations was higher for severe anemia compared to mild/moderate anemia (Additional file 1: Fig. S3). Rates of adverse events associated with anemia remained elevated across categories of sex, ejection fraction, and presence of diabetes, and after accounting for death as a competing risk (Additional file 1: Tables S10 and S11).

**Table 3:** Adverse clinical outcomes associated with developing anemia in patients with heart failure

Outcomes	Exposure	No. of patients/events	Incidence rate (95% CI), per 1000 person-years	Adjusted hazard ratio (95% CI)
***Main analysis***				
All-cause death	No anemia	8932/1947	74.31 (71.08–77.65)	Reference
	Anemia	3049/1505	215.05 (204.46–226.05)	2.04 (1.82–2.29)
MACE	No anemia	8932/1454	57.87 (54.97–60.88)	Reference
	Anemia	3049/982	153.15 (143.87–162.87)	2.13 (1.85–2.44)
HHF	No anemia	8932/1780	76.37 (72.90–79.96)	Reference
	Anemia	3049/1397	285.45 (270.87–300.61)	4.85 (4.30–5.48)
Cancer	No anemia	8932/1505	64.95 (61.75–68.27)	Reference
	Anemia	3049/940	171.21 (160.62–182.33)	3.41 (3.09–3.77)
***Sensitivity analysis excluding events occurring within the first 180 days***				
All cause-death	No anemia	8932/1947	74.31 (71.08–77.65)	Reference
	Anemia	3049/1183	169.04 (159.68–178.8)	1.58 (1.40–1.79)
MACE	No anemia	8932/1454	57.87 (54.97–60.88)	Reference
	Anemia	3049/677	105.58 (97.93–113.68)	1.37 (1.18–1.59)
HHF	No anemia	8932/1780	76.37 (72.90–79.96)	Reference
	Anemia	3049/628	128.32 (118.67–138.55)	2.02 (1.75–2.33)
Cancer	No anemia	8932/1505	64.95 (61.75–68.27)	Reference
	Anemia	3049/410	74.68 (67.8–82.08)	1.35 (1.19–1.54)

Abbreviations: *CI*, confidence interval; *HF*, heart failure; *HR*, hazard ratio; *MACE*, major adverse cardiovascular event; *HHF*, hospitalization for heart failure

Note: MACE was defined as a composite of nonfatal stroke, nonfatal myocardial infarction, and cardiovascular death. The analyses for all adverse outcomes were adjusted for age, sex, education level, calendar year, baseline estimated glomerular filtration rate, baseline hemoglobin, diabetes, hypertension, ischemic heart disease, cerebrovascular disease (includes stroke), peripheral vascular disease, atrial fibrillation, valve disease, chronic obstructive pulmonary disease, rheumatoid diseases, dementia, liver disease, peptic ulcer disease, melanoma, device therapies, ACE inhibitors/angiotensin II receptor blockers (ARBs), beta-blockers, calcium channel blockers, loop diuretics, mineralocorticoid receptor antagonists, digoxin, statins, platelet inhibitors, anticoagulants, non-steroidal anti-inflammatory drugs, other blood pressure medications

## Discussion

This study provides a comprehensive assessment for the clinical work-up of de novo anemia in ambulatory patients with HF. Compared with previous studies, we overcome limitations of prevalent anemia designs and provide larger precision and granularity, expanding beyond testing for iron stores and evaluating quality indicators across more severe anemias and settings of care. We find that the clinical work-up of patients with HF and incident anemia was suboptimal: only 1 in 7 anemia events received a clinical diagnosis, less than half underwent laboratory testing for iron stores or received treatment, and around 1 in 6 had procedural screening for bleeding or cancer. Clinical work-up was more extensive among patients with severe anemias, but still insufficient: about half of severe anemias received a clinical diagnosis, and 25–35% of those did not receive treatment or were screened for iron deficiency. Although time trends show a modest improvement over time, this study provides evidence of a clinical gap and suggests uncertainty regarding work-up and treatment of anemia in HF.

Anemia is clearly common in HF. In our study, 38.3% of patients had anemia at cohort inclusion (i.e. prevalent anemia cases) and 34.1% additional patients developed incident anemia during a median of 2.7 years follow-up. In a previous study from the US Cardiovascular Research Network (CVRN) collecting routine data from 2005 to 2012, the incidence of anemia in patients with HF was higher than in our study, reaching up to 57.1% of cases [5]. Differences between our observations may represent true country-to-country differences, or discordances in the models of care (pay-for-service versus universal heathcare model), but they may also likely be attributed to differences in the periods evaluated, particularly before clinical guidelines recognized the commonness of both anemia and iron deficiency, and the need to screen for and treat iron deficiency. Our multivariable risk analysis confirms the diversity of the risk factors likely involved in anemia, which in agreement with previous studies [18, 19] include older age, male sex, use of loop diuretics, diabetes mellitus, and chronic kidney disease.

Our key finding is that the work-up and management of anemia in HF appears to be under-addressed. A low proportion (15.7%) of anemia events were accompanied by a clinical diagnosis, and about 50% of cases of severe anemia lacked a diagnosis. These low rates of recognition may reflect suboptimal awareness of the importance of detecting and managing anemia. Our findings highlight the gap between ESC guideline recommendations and real-world practice. The ESC guidelines advocate systematic iron testing and intravenous iron supplementation for symptomatic HFrEF/HFmrEF patients with iron deficiency [7, 20]. However, only 44.8% of anemia cases underwent iron testing, and merely 16.1% received intravenous iron in our study, indicating underutilization of guideline-directed therapies. This discrepancy may stem from fragmented care across specialties or limited awareness of recent evidence. Future health planning should prioritize protocolized screening for iron deficiency and use of intravenous iron, particularly in severe anemia, to align with guideline goals of reducing hospitalizations and improving patients’ quality of life. While the testing for other routine biomarkers in the clinical work-up of anemia was high, testing for iron stores was less common (in < 50% of all anemia cases, and 65% of all severe anemia cases). Our findings agree with a previous U.S. study [5] and conflict with current guideline recommendations [20, 21]. Interestingly, testing rates did not meaningfully differ between primary care or cardiologist-specialist care. In agreement with our previous observation of improved iron screening rates at HF diagnosis [8], our time-trend analysis indicates a gradual improvement in the iron screening rates during anemia diagnostic work-up. This is a positive improvement, given the low cost of iron tests and that iron deficiency is easily mitigated through oral or intravenous iron. However, on the other hand, concern for and treatment of iron deficiency may detract attention from or even mask other causes of anemia, such as screening for bleeding or cancer.

In clinical practice, the management of anemia in HF patients necessitates a balanced approach that integrates guideline recommendations with individualized patient assessment. The American Gastroenterological Association (AGA) Clinical Practice Guidelines advocate for bidirectional endoscopy in patients with unexplained iron deficiency anemia, particularly those with gastrointestinal symptoms, to exclude occult bleeding or malignancy [22]. Similarly, the National Institute for Health and Care Excellence (NICE) Suspected Cancer Guideline from the UK supports risk-stratified cancer assessment in patients aged ≥ 50 years with unexplained anemia and alarm symptoms [23]. The KDIGO Anemia Guideline further emphasizes the importance of excluding bleeding sources before initiating therapy in chronic kidney disease (CKD)-related anemia [24]. Our study shows that in a few cases, other potential causes of anemia and iron deficiency were investigated. Future management of anemia or iron deficiency in HF patients might consider the guidelines recommendation from other specialties to rule out or initiate the differential diagnosis of underlying cancer or bleeding.

Another key observation of our study is that once anemia was detected and proved to be sustained for at least 6 months, only about 40% of patients received treatment for it. This low rate of treatment may reflect the perception that mild-to-moderate anemias (37.7% of which received treatment) may not be as worthy of treatment as severe anemias (treated in 76.6% of cases). The most common treatment strategies to manage anemia in our study were iron replacement therapy (both oral and intravenous). A previous analysis from the UK showed that only 7% of patients with new-onset anemia received oral iron therapy, which is even lower treatment rates than our study [17]. This may reflect the poor absorption and poor effectiveness of oral iron therapy, particularly in patients with comorbidities such as HF. Notably, treatment rates were higher in cardiology care (43.0%) versus primary care (29.4%), which may reflect both guideline adherence patterns and patient severity stratification. Cardiologists may more often comply with the ESC recommendations. However, our observational study may also reflect that more severe anemia cases (e.g., hemoglobin < 10 g/dL) end up being referred to and managed by specialists [3], or that intravenous iron is more frequently administered in specialized settings due to concerns of safety and monitoring requirements [25]. Further, referral bias may lead to more severe anemia cases being managed by cardiologists [5]. This may reflect concerns about thromboembolic risks, as demonstrated in the RED-HF trial, where targeting hemoglobin to 13 g/dL with darbepoetin alfa in systolic heart failure increased thromboembolic events without improving outcomes [26]. While direct evidence for the optimal level of hemoglobin in patients with HF is lacking, data from CKD populations demonstrate a clear hemoglobin-dependent risk gradient. Earlier CKD trial data (CHOIR [27]; CREATE [28]) confirmed heightened thrombotic risk above 11.5 g/dL. Collectively, the evidence supports adopting a conservative hemoglobin target (10–11.5 g/dL) to mitigate ESA-associated risks in CKD populations [27–29]. Until prospective HF-specific studies are available, extrapolation from CKD evidence supports adopting a conservative hemoglobin target (10–11.5 g/dL) to mitigate ESA-associated thrombotic event risks in this population. Time trends suggest improved rates of treatment over time, and the proportion of treated anemias was higher in patients managed in cardiology care (43% of cases treated) compared to those managed in primary care (29% of cases treated).

New-onset anemia identifies a group of patients at high risk for poor outcomes. Our observation that patients who developed anemia were at higher subsequent risk of death or CVD events was expected and has been reported before [5]. Until further evidence is provided, these associations are likely to be explained by anemia being a marker for the presence of more severe systemic disease given the inconsistencies in trials regarding the effect of anemia or iron deficiency correction on death or HF readmission [25, 30]. Notwithstanding, anemia is still an important marker for the risk of adverse events whose correction has demonstrated to improve functional status and quality of life [21, 31]. Our study also observed an increased risk of cancer, which agrees with previous studies as well [17, 20, 31–33]. We note that apart from basic laboratory testing, diagnostic work-up for underlying cancer was rare. While there may be many appropriate reasons to refrain from invasive testing, it would seem low that only 4% and 10% of anemia cases in our study were candidates for colonoscopy and esophagogastroduodenoscopy, respectively.

The strengths of our study include a focus on incident anemia and complete capture of care processes in a single large region with universal health care. This circumvents some limitations of previous studies, including their cross-sectional design and fragmentation of patient cohorts between care and private care practices. We were able to quantify with precision the diagnostic work-up and follow these processes across cardiologists vs. primary care practitioners. The limitations of our study include a reliance on ICD codes for some medical diagnoses, which can provide inaccurate outcome measures. We represent clinical practice in Stockholm between 2016 and 2021. Caution is advised for extrapolation to other periods, regions, or countries with limited health care resources. As an additional limitation, we were not able to identify patients receiving palliative care, which may have influenced the decision to treat or not the identified anemia. Patients may have bought over-the-counter oral iron, and this would not be captured by our study, but we estimate that the possibility of effective self-medication was low. Further, we compare whether clinical work-ups, especially the iron tests, align with guideline recommendations, but we cannot evaluate with certainty whether the reactions were or were not appropriate; in multimorbid patients or end-of-life care, for instance, clinicians may prioritize managing other conditions over anemia. Data on other processes related to the work-up of anemia, such as fecal occult blood testing, were not available. Lack of the results of esophagogastroduodenoscopy or colonoscopy examinations limits our ability to confirm the benefits of such screening to rule out undergoing cancer or bleeding. As an observational study, we were not able to further explore the causal relationship between iron supplementation (oral or intravenous) and clinical outcomes in HF patients. Available randomized controlled trials show somewhat inconsistent effects of iron repletion in patients with HF [25, 30]. We did not have ejection fraction measures, but instead estimated ejection fraction categories by an externally validated Swedish algorithm. As in all estimations, there is a possibility of misclassification bias of HF phenotype. Information on BMI or smoking habits was not available in our data source, potentially introducing unmeasured confounding.

## Conclusions

This study shows that incident anemia is a common occurrence among patients with HF. However, anemia events in this high-risk population were not sufficiently recognized and cared for, as evidenced by a low proportion of issued diagnoses, low rates of screening for iron stores or invasive testing for malignancy, and low rates of treatment. Finally, incident anemia was associated with a considerably increased subsequent risk of MACE, HF hospitalization, cancer and death. While anemia epidemiology and work-up priorities may differ from other populations (e.g., cancer or CKD), our findings highlight gaps in HF care that warrant correction.

## Supplementary Information

Additional file 1: Method S1. Ejection fraction prediction algorithm in patients with heart failure. Table S1. Variables used for inclusion and exclusion criteria. Table S2. Definition of study outcomes. Table S3. Definition of clinical work-up of incident anemia. Table S4. Definitions for covariates including demographic characteristics, comorbidities, medications, undertaken surgical procedures and laboratory tests. Table S5. Baseline covariates considered in the analysis of conditions associated with incident anemia. Table S6. Incidence rates of anemia in patients with heart failure. Table S7. Clinical work-up of anemia stratified by the presence/absence of concurrent iron testing. Table S8. Clinical work-up of anemia by settings of management. Table S9. Clinical work-up of anemia after excluding patients died in the first 6 to 12 month after incident anemia. Table S10. Subgroup analyses: Adjusted hazard ratios of incident anemia and study outcomes. Table S11. Comparison of traditional and competing risk models for study outcomes. Figure S1. Patient selection flowchart. Figure S2. Strength of the multivariable association between baseline conditions associated with incident anemia. Figure S3. Adverse clinical outcomes associated developing anemia in patients with heart failure.

## Data Availability

The datasets used and/or analysed during the current study are available from the corresponding author on reasonable request.

## Abbreviations

ACE: Angiotensin-converting enzyme
ALT: Alanine aminotransferase
ARB: Angiotensin II receptor blockers
AST: Aspartate aminotransferase
CI: Confidence interval
CKD: Chronic kidney disease
CRP: C-reactive protein
CVD: Cerebrovascular disease
eGFR: Estimated glomerular filtration rate
ESA: Erythropoietin stimulating agents
ESC: European Society of Cardiology
HF: Heart failure
HFpEF: Heart failure with preserved ejection fraction
HFrEF: Heart failure with reduced ejection fraction
HR: Hazard ratio
ICD: International Classification of Diseases
IQR: Interquartile range
MACE: Major adverse cardiovascular events
MRA: Mineralocorticoid receptor antagonists
SD: Standard deviation
TSAT: Transferrin saturation
RAS: Renin–angiotensin system
WHO: World Health Organization
