# Supplementary material for: Diagnostic work-up of anemia and associated health outcomes in people with heart failure
Source: BMC Med. 2025 Aug 12;23:471. doi: 10.1186/s12916-025-04303-8 (PMC12345016; doi:10.1186/s12916-025-04303-8)
Supplement: Supplementary file 1 — Additional file 1: Method S1. Ejection fraction prediction algorithm in patients with heart failure. Table S1. Variables used for inclusion and exclusion criteria. Table S2. Definition of study outcomes. Table S3. Definition of clinical work-up of incident anemia. Table S4. Definitions for covariates including demographic characteristics, comorbidities, medications, undertaken surgical procedures and laboratory tests. Table S5. Baseline covariates considered in the analysis of conditions associated with incident anemia. Table S6. Incidence rates of anemia in patients with heart failure. Table S7. Clinical work-up of anemia stratified by the presence/absence of concurrent iron testing. Table S8. Clinical work-up of anemia by settings of management. Table S9. Clinical work-up of anemia after excluding patients died in the first 6 to 12 month after incident anemia. Table S10. Subgroup analyses: Adjusted hazard ratios of incident anemia and study outcomes. Table S11. Comparison of traditional and competing risk models for study outcomes. Figure S1. Patient selection flowchart. Figure S2. Strength of the multivariable association between baseline conditions associated with incident anemia. Figure S3. Adverse clinical outcomes associated developing anemia in patients with heart failure. [file 12916_2025_4303_MOESM1_ESM.docx]

**Additional file 1**

**Diagnostic work-up of anemia and associated health outcomes in people with heart failure.**

Authors: Guobin Su, Ruowei Xiao, Dongze Ji, Kaiyu He, Anna Hallert, Gianluigi Savarese, Lars H. Lund, Yang Xu, Juan Jesus Carrero

[**Method S1.** Ejection fraction prediction algorithm in patients with heart failure^1^ 2](#_Toc203665165)

[**Table S1.** Variables used for inclusion and exclusion criteria 3](#_Toc203665166)

[**Table S2.** Definition of study outcomes 4](#_Toc203665167)

[**Table S3.** Definition of clinical work-up of incident anemia 5](#_Toc203665168)

[**Table S4.** Definitions for covariates including demographic characteristics, comorbidities, medications, undertaken surgical procedures and laboratory tests 6](#_Toc203665169)

[**Table S5.** Baseline covariates considered in the analysis of conditions associated with incident anemia 10](#_Toc203665170)

[**Table S6.** Incidence rates of anemia in patients with heart failure 11](#_Toc203665171)

[**Table S7.** Clinical work-up of anemia stratified by the presence/absence of concurrent iron testing 12](#_Toc203665172)

[**Table S8.** Clinical work-up of anemia by settings of management 13](#_Toc203665173)

[**Table S9.** Clinical work-up of anemia after excluding patients died in the first 6 to 12 month after incident anemia. 15](#_Toc203665174)

[**Table S10.** Subgroup analyses: Adjusted hazard ratios of incident anemia and study outcomes 16](#_Toc203665175)

[**Table S11.** Comparison of traditional and competing risk models for study outcomes 17](#_Toc203665176)

[**Figure S1.** Patient selection flowchart 18](#_Toc203665177)

[**Figure S2.** Strength of the multivariable association between baseline conditions associated with incident anemia 19](#_Toc203665178)

[**Figure S3.** Adverse clinical outcomes associated developing anemia in patients with heart failure 21](#_Toc203665179)

### **Method S1.** Ejection fraction prediction algorithm in patients with heart failure^1^

Simplified EF algorithm (without NYHA or NT-proBNP)

For all patients with heart failure at baseline, EF can be predicted with the following formula:

$$P\left( EF\geq50\% \right)=\frac{X}{1+X}$$

With

$X=exp(\ln\left( 0.32 \right)+\mathbb{1}_{age\geq75}*\ln\left( 1.58 \right)+\mathbb{1}_{sex=female}*\ln\left( 2.33 \right)+\mathbb{1}_{eGFR<30}*\ln\left( 0.60 \right)+\mathbb{1}_{eGFR 30-60}*\ln\left( 0.82 \right)+\mathbb{1}_{eGFR 60-90}*\ln\left( 0.87 \right)+\mathbb{1}_{ischaemic heart disease=yes}*\ln\left( 0.63 \right)+\mathbb{1}_{anemia=yes}*\ln\left( 1.44 \right)+\mathbb{1}_{atrial fibrillation=yes}*\ln\left( 1.45 \right)+\mathbb{1}_{COPD=yes}*\ln\left( 1.34 \right)+\mathbb{1}_{diabetes=yes}*\ln\left( 1.01 \right)+\mathbb{1}_{hypertension=yes}*\ln\left( 1.68 \right)+\mathbb{1}_{valvular disease=yes}*\ln\left( 1.31 \right)+\mathbb{1}_{malignant cancer=yes}*\ln\left( 1.18 \right)+\mathbb{1}_{device therapy=yes}*\ln\left( 0.30 \right)+\mathbb{1}_{RAS-inhibitor use=yes}*\ln\left( 0.40 \right)+\mathbb{1}_{beta-blocker use=yes}*\ln\left( 0.51 \right)+\mathbb{1}_{MRA use=yes}*\ln\left( 0.78 \right)+\mathbb{1}_{digoxin use=yes}*\ln\left( 0.91 \right)+\mathbb{1}_{diuretic use=yes}*\ln\left( 1.15 \right))$

Hereby, $\mathbb{1}$ is the indicator function, meaning that:

$$\mathbb{1}_{age\geq75}= \left\{ \begin{aligned} 1, if age\geq75 \\ 0, if age<75 \end{aligned} \right.$$

Cut-off

A predicted probability threshold cut-off of 0.23 can be used to maximize the sensitivity and specificity of the model, as previously defined, creating the following EF phenotypes:

$$Predicted EF phenotype at baseline= \left\{ \begin{aligned} no HF at baseline ,if no HF at baseline \\ HFrEF or HFmrEF (predicted EF<50\%), if P\left( EF\geq50\% \right)<0.23 \\ HFpEF \left( predicted EF\geq50\% \right), , if P\left( EF\geq50\% \right)\geq0.23 \end{aligned} \right.$$

Predictors in the model are: age (categorized as <75 and ≥75 years), sex (male/female), clinical characteristics: eGFR (<30, 30-60, 60-90 and ≥90 ml/min/1.73m2), comorbidities (yes vs. no), history of ischaemic heart disease, atrial fibrillation, chronic obstructive pulmonary disease [COPD], diabetes, hypertension, anemia, cancer in the previous 3 years, valvular disease, and use of treatments (yes vs. no), device therapy [implantable cardioverter defibrillator or cardiac resynchronization therapy], renin–angiotensin system [RAS] inhibitors, beta-blockers, diuretics, mineralocorticoid receptor antagonist [MRA], digoxin.

Use of medication were defined as presence of prescription 6 months before the index date.

**Reference**

[1] Uijl A, Lund LH, Vaartjes I, et al. A registry-based algorithm to predict ejection fraction in patients with heart failure. *ESC Heart Fail*. 2020;7(5):2388-2397. doi:10.1002/ehf2.12779

### **Table S1.** Variables used for inclusion and exclusion criteria

| **Eligibility criteria** | **ICD-10/** **KVÅ /ATC codes** |
| --- | --- |
| ***Inclusion criteria*** |  |
| Heart failure | ICD-10 codes: I50, I110, I113, I971, K761, P290 |
| ***Exclusion criteria*** |  |
| ***for non-eligible Hb tests*** |  |
| Bleeding | Intracranial bleeding, ICD-10 codes: I60-62, S064-066, I690-692  Gastrointestinal bleeding, ICD-10 codes: I850, I983, K226, K250, K252, K254, K256, K260, K262, K264, K266, K270, K272, K274, K276, K280, K284, K286, K290, K625, K661, K920, K921, K922  Urogenital bleeding, ICD-10 codes: N02, R319, N95  Other bleeding, ICD-10 codes: H431, R04, R58, D629, or KVÅ code: DR029 |
| Transfusions | ICD-10 codes: Z513  KVÅ codes: DR029, DR030, DR033, DR034 |
| ***for non-eligible patients*** |  |
| Recent pregnancy or childbirth (previous 2 years) | ICD-10 codes: O00-O99 |
| Cancer (previous 3 years) | ICD-10 codes: C00–C99(except C43, C44) |
| Hematological diseases | ICD-10 codes: D75 |
| Hereditary hematological disease | ICD-10 codes: D56, D57 |
| Chronic infections (hepatitis, tuberculosis or HIV) | ICD-10 codes: A15-A19, B15-B24 |
| Inflammatory bowel disease | ICD-10 codes: K50-K51 |
| Baseline anemia | a low Hb value by WHO definition: Hb <13 g/dL for men or < 12 g/dL for women at the index date, or  anemia diagnosis (ICD-10 codes: D60-64), or  treatment of anemia (iron oral, ATC codes: B03A, or iron intravenous, KVÅ code: DT016 or ESA, ATC codes: B03X) in the year prior to the index date |

**Abbreviations**: ATC, Anatomical Therapeutic Chemical; ESA, erythropoietin-stimulating agent; Hb, hemoglobin; HIV, human immunodeficiency virus; ICD-10, International Classification of Diseases 10th Revision; KVÅ, Klassifikation av vårdåtgärder, a Swedish procedure coding system, including medical procedures (KMÅ) and surgical procedures (KKÅ); WHO, World Health Organization.

### **Table S2.** Definition of study outcomes

| Outcome | Definition |
| --- | --- |
| *Analysis 1* |  |
| Anemia | An Hb measurement <12 g/dL for women or <13 g/dL for men, followed by a diagnosis of anemia (ICD-10 codes: D60-D64), or treatment initiation (iron oral, ATC codes: B03A, or iron intravenous, KVÅ codes: DT016 or ESA, ATC codes: B03X) within 3 months, or a subsequent Hb measurement with similar magnitude between 3 to 6 months apart. |
| Mild/moderate | An Hb measurement <12 g/dL for women or <13 g/dL for men but ≥10 g/dL, followed by a diagnosis of anemia (ICD-10 codes: D60-D64), or treatment initiation (iron oral, ATC codes: B03A, or iron intravenous, KVÅ codes: DT016 or ESA, ATC codes: B03X) within 3 months, or a second Hb measurement with similar magnitude between 3 to 6 months apart. |
| Severe | An Hb measurement <10 g/dL regardless of sex, followed by a diagnosis of anemia (ICD-10 codes: D60-64), or treatment initiation (iron oral, ATC codes: B03A, or iron intravenous, KVÅ codes: DT016 or ESA, ATC codes: B03X) within 3 months, or a second Hb measurement <10 g/dL between 3 to 6 months apart. |
| *Analysis 2* |  |
| All-cause death | Death by any cause |
| MACE | Cardiovascular death: death attributed to ICD-10 codes G45-G46, H341 or I  Non-fatal myocardial infarction: diagnosis with ICD-10 codes I21-I23  Non-fatal stroke: diagnosis with ICD-10 codes H341, G45-G46, I60-I61, I63-I64 |
| Hospitalized heart failure | Inpatient diagnosis of heart failure with ICD-10 codes I099, I110, I130, I132, I255, I420, I425-I429, I43, I50, P290 |
| Cancer | Diagnosis of all ICD C category |

**Abbreviations**: ATC, Anatomical Therapeutic Chemical; CVD, cardiovascular disease; ESA, erythropoietin-stimulating agent; ICD-10, International Classification of Diseases 10^th^ Revision; MACE, major adverse cardiovascular event; KVÅ, Klassifikation av vårdåtgärder, a Swedish procedure coding system, including medical procedures (KMÅ) and surgical procedures (KKÅ).

### **Table S3.** Definition of clinical work-up of incident anemia

| Clinical work-up | Definition |
| --- | --- |
| **Anemia recognition** | A clinical diagnosis of anemia (ICD-10 codes: D60-D64) within 6 months from the incident anemia event. |
| **Testing for iron stores** | A laboratory test of ferritin or TSAT within 6 months from the incident anemia event. |
| **Other laboratory testing** |  |
| Liver enzymes (ALT or AST) | A laboratory test of ALT or AST within 6 months from the incident anemia event. |
| Serum/plasma creatinine | A laboratory test of serum/plasma creatinine within 6 months from the incident anemia event. |
| Inflammation test | A laboratory test of CRP within 6 months from the incident anemia event. |
| **Procedures for detection of bleeding or cancer** |  |
| Colonoscopy | A test of colonoscopy (KVÅ codes: UJF32) within 6 months from the incident anemia event. |
| Urinalysis | A test of urinalysis (KVÅ codes: UJF35) within 6 months from the incident anemia event. |
| Cystoscopy | A test of cystoscopy (KVÅ codes: UKC02) within 6 months from the incident anemia event. |
| Esofagogastroduodenoscopy | A test of esofagogastroduodenoscopy (KVÅ codes: UJC02, UJC05, UJC12, UJC15, UJD02, UJD05) within 6 months from the incident anemia event. |
| **Initiation of treatments** | Treatments of anemia within 6 months from the incident anemia event, including blood transfusions (ICD-10 codes: Z513; KVÅ codes: DR029, DR030, DR033, DR044), iron intravenous (Kvå code: DT016), oral iron(ATC codes: B03A) and ESAs (ATC codes: B03X). |

**Abbreviations**: ALT, Alanine aminotransferase; AST, Aspartate aminotransferase; CRP, C-reactive protein; ESA, erythropoietin-stimulating agent; ICD-10, International Classification of Diseases 10^th^ Revision; TSAT, transferrin saturation; KVÅ, Klassifikation av vårdåtgärder, a Swedish procedure coding system, including medical procedures (KMÅ) and surgical procedures (KKÅ).

### **Table S4.** Definitions for covariates including demographic characteristics, comorbidities, medications, undertaken surgical procedures and laboratory tests

| **Covariates** | **ICD-10/** **KVÅ /ATC codes** | **Time windows** | **Missing rate at baseline, *n (%)*** |
| --- | --- | --- | --- |
| ***Demographic characteristics*** |  |  |  |
| Age | / | At the index date | 0(0) |
| Sex | / | At the index date | 0(0) |
| Educational | / | At the index date | 302 (3.4) |
| Canlendar year | / | At the index date | 0(0) |
| ***Comorbid conditions*** |  |  |  |
| Diabetes mellitus | ICD-10 codes: E10-E14 | Any time before index date and updated at the time of incident anemia occurrence | 0(0) |
| Hypertension | ICD-10 codes: I10-I15 | Any time before index date and updated at the time of incident anemia occurrence | 0(0) |
| Ischemic heart disease | ICD-10 codes: I20, I21, I22, I23, I24, I25 | Any time before index date and updated at the time of incident anemia occurrence | 0(0) |
| Cerebrovascular disease (includes stroke) | ICD-10 codes: G45-46, H340, I60-69 | Any time before index date and updated at the time of incident anemia occurrence | 0(0) |
| Peripheral vascular disease | ICD-10 codes: I70-71, I731, I738, I739, I771, I790, I792, K551, K558, K559, Z958, Z959 | Any time before index date and updated at the time of incident anemia occurrence | 0(0) |
| Atrial fibrillation | ICD-10 codes: I48 | Any time before index date and updated at the time of incident anemia occurrence | 0(0) |
| Valve disease | ICD-10 codes: I05, I06, I07, I08, I35 | Any time before index date and updated at the time of incident anemia occurrence | 0(0) |
| Chronic obstructive pulmonary disease | ICD-10 codes: J40-J47 | Any time before index date and updated at the time of incident anemia occurrence | 0(0) |
| Rheumatoid disease | ICD-10 codes: L40, L93, M05, M06, M10, M140, M315, M353, M32, M34 | Any time before index date and updated at the time of incident anemia occurrence | 0(0) |
| Dementia | ICD-10 codes: F00-F03, F05.1, G30, G311 | Any time before index date and updated at the time of incident anemia occurrence | 0(0) |
| Liver disease | ICD-10 codes: K70-77 | Any time before index date and updated at the time of incident anemia occurrence | 0(0) |
| Peptic ulcer disease | ICD-10 codes: K27 | Any time before index date and updated at the time of incident anemia occurrence | 0(0) |
| Melanoma | ICD-10 codes: C43, C44 | In the 3 years before index date and updated at the time of incident anemia occurrence | 0(0) |
| ***Medications and undertaken surgical procedures*** | | |  |
| Device therapies | ICD-10 codes: Z950, Z95810, Z450; KVÅ codes: FPE, FPF, FPG, DF016 | In the 6 months before index date and updated at the time of incident anemia occurrence | 0(0) |
| Angiotensin-converting enzyme (ACE) inhibitors/ angiotensin II receptor blockers (ARBs) | ATC codes: C09A–C09D | In the 6 months before index date and updated at the time of incident anemia occurrence | 0(0) |
| Beta-blockers | ATC codes: C07 | In the 6 months before index date and updated at the time of incident anemia occurrence | 0(0) |
| Calcium channel blockers | ATC codes: C08C–C08D | In the 6 months before index date and updated at the time of incident anemia occurrence | 0(0) |
| Loop diuretics | ATC codes: C03C, C03EB | In the 6 months before index date and updated at the time of incident anemia occurrence | 0(0) |
| Mineralocorticoid receptor antagonists (MRAs) | ATC codes: C03DA | In the 6 months before index date and updated at the time of incident anemia occurrence | 0(0) |
| Digoxin | ATC codes: C01AA05 | In the 6 months before index date and updated at the time of incident anemia occurrence | 0(0) |
| Statins | ATC codes: C10AA, C10B | In the 6 months before index date and updated at the time of incident anemia occurrence | 0(0) |
| Immunosuppressants | ATC codes: L04 | In the 6 months before index date and updated at the time of incident anemia occurrence | 0(0) |
| Platelet aggregation inhibitors | ATC codes: B01AC | In the 6 months before index date and updated at the time of incident anemia occurrence | 0(0) |
| Anticoagulants except heparin | ATC codes: B01AA, B01AE, B01AF, B01AX | In the 6 months before index date and updated at the time of incident anemia occurrence | 0(0) |
| Non-steroid anti-inflammatory drugs | ATC codes: M01A | In the 6 months before index date and updated at the time of incident anemia occurrence | 0(0) |
| Other blood pressure medications | ATC codes: C02, C08E, C08G | In the 6 months before index date and updated at the time of incident anemia occurrence | 0(0) |
| ***Laboratery tests*** | | |  |
| Hb | / | At the index date and updated at the time of incident anemia occurrence | 0(0) |
| Creatinine-based eGFR | / | Closest to the index date and up to one year before the index date and updated at the time of incident anemia occurrence | 173 (1.9) |

**Abbreviations**: ATC, Anatomical Therapeutic Chemical; eGFR: estimated glomerular filtration rate using the 2008 CKD-EPI equation without correction for race; Hb, hemoglobin; ICD-10, International Classification of Diseases 10th Revision; KVÅ, a Swedish procedure coding system, including medical procedures (KMÅ) and surgical procedures (KKÅ).

### **Table S5.** Baseline covariates considered in the analysis of conditions associated with incident anemia

| **Predictors** |  |
| --- | --- |
| Demographic characteristics | Age at baseline, sex |
| Heart failure type | HFpEF: EF≥50%/HFrEF: EF<50% |
| Comorbidities | Diabetes mellitus, hypertension, ischemic heart disease, peripheral vascular disease, cerebrovascular disease (includes stroke), atrial fibrillation, valve disease, chronic obstructive pulmonary disease, dementia, rheumatoid diseases, liver disease, peptic ulcer disease, melanoma |
| Medications or treatments | Device therapies, ACEIs/ARBs, beta blockers, calcium channel blockers, loop diuretics, MRA, digoxin, statins, immunosuppressant, platelet inhibitors, anticoagulants, NSAIDs, other blood pressure medications |
| Laboratory tests | Hb, eGFR |

**Abbreviations**: ACEI, angiotensin-converting enzyme inhibitor; ARB, angiotensin II receptor blocker; eGFR, estimated glomerular filtration rate; Hb, hemoglobin; MRA, mineralocorticoid receptor antagonists; NSAID, non-steroidal anti-inflammatory drug; TSAT, transferrin saturation.

### **Table S6.** Incidence rates of anemia in patients with heart failure

|  | **No. of patients** | **No. of events** | **Median follow-up [IQR], years** | **Incidence rate per 1000 person-years (95% CI)** |
| --- | --- | --- | --- | --- |
| Any anemia | 8932 | 3049 | 2.7 [1.1-4.9] | 116.4 (97.1-138.5) |
| Mild/moderate anemia | 8932 | 2970 | 2.7 [1.1-4.9] | 112.7 (93.8-134.4) |
| Severe anemia | 8932 | 1167 | 3.7 [1.9-5.3] | 37.4 (27.2-50.3) |

**Abbreviations**: CI, confidence interval; IQR, interquartile range.

**Note**: Anemia was defined as a low Hb measurement (World Health Organization definition: <12 g/dL for females or <13 g/dL for males) followed by a diagnosis of anemia (International Classification of Diseases 10^th^ Revision codes D60–64 or treatment initiation within 3 months, or a subsequent Hb measurement with similar magnitude between 3 and 6 months apart (i.e., anemia sustained for at least 3 months). Mild/moderate anemia was defined as a Hb measurement <12 g/dL for females or <13 g/dL for males but ≥10g/dL, and severe anemia was defined as Hb <10 g/dL regardless of sex.

### **Table S7.** Clinical work-up of anemia stratified by the presence/absence of concurrent iron testing

|  | **With iron testing** | | | **Without iron testing** |
| --- | --- | --- | --- | --- |
|  | **Overall** | **No iron deficiency** | **Iron deficiency** |  |
| No. of anemia cases | 1356 | 485 | 880 | 1684 |
| Received an anemia diagnosis, *n (%)* | 330 (24.2) | 113 (23.3) | 217(24.7) | 150 (8.9) |
| Other recommend laboratory testing, *n (%)* | | | | |
| Liver enzymes (ALT or AST) | 1184(86.7) | 423 (87.2) | 761 (86.5) | 1338 (79.5) |
| Serum/plasma creatinine | 1363(99.9) | 485 (100) | 878 (99.8) | 1680 (99.8) |
| CRP | 1253(91.8) | 455 (93.8) | 798 (90.7) | 1536 (91.2) |
| Other recommend procedures, *n (%)* | | | | |
| Colonoscopy | 94 (6.9) | 23 (4.7) | 71 (8.1) | 36 (2.1) |
| Urinalysis | 43 (3.2) | 16 (3.3) | 27 (3.1) | 18 (1.1) |
| Cystoscopy | 63 (4.6) | 24 (4.9) | 39 (4.4) | 101 (6.0) |
| Esofagogastroduodenoscopy | 206 (15.1) | 67 (13.8) | 139 (15.8) | 107 (6.4) |
| Treatment patterns, *n (%)* |  |  |  |  |
| Iron (oral or intravenous) | 486 (35.6) | 127 (26.2) | 359 (40.8) | 357 (21.2) |
| Blood transfusion | 77 (5.6) | 40 (8.2) | 37 (4.2) | 107 (6.4) |
| ESA | 2 (0.1) | 1 (0.2) | 1 (0.1) | 0 (0.0) |
| Combination treatment | 100 (7.3) | 28 (5.8) | 72 (8.2) | 60 (3.6) |
| Iron + blood transfusion | 90 (6.6) | 26 (5.4) | 64 (7.3) | 60 (3.6) |
| Iron + ESA | 5 (0.4) | 1 (0.2) | 4 (0.5) | 0 (0.0) |
| Blood transfusion + ESA | 3 (0.2) | 1 (0.2) | 2 (0.2) | 0 (0.0) |
| Iron + blood transfusion + ESA | 2 (0.1) | 0 (0.0) | 2 (0.2) | 0 (0.0) |
| Total population receiving treatment | 665 (48.7) | 196 (40.4) | 469 (53.3) | 524 (31.1) |

**Abbreviations**: ALT, Alanine aminotransferase; AST, Aspartate aminotransferase; CRP, C-reactive protein; ESA, erythropoietin-stimulating agent; intravenous; TSAT, transferrin saturation.

### **Table S8.** Clinical work-up of anemia by settings of management

|  | **Primary care** | | | **Cardiology care** | | | **Other care** | | |
| --- | --- | --- | --- | --- | --- | --- | --- | --- | --- |
|  | **Any anemia** | **Mild/moderate**  **anemia** | **Severe anemia** | **Any anemia** | **Mild/moderate**  **anemia** | **Severe anemia** | **Any anemia** | **Mild/moderate**  **anemia** | **Severe anemia** |
| No. of anemia cases | 619 | 602 | 126 | 1487 | 1464 | 577 | 943 | 904 | 464 |
| Received an anemia diagnosis, *n (%)* | 88 (14.2) | 84(14.0) | 55(43.7) | 234 (15.7) | 215(14.7) | 282(48.9) | 158(16.8) | 132(14.6) | 241(51.9) |
| Tested for iron stores, *n (%)* | 293(47.3) | 281(46.7) | 94(74.6) | 731 (49.2) | 713(48.7) | 398(69.0) | 341(36.2) | 318(35.2) | 270(58.2) |
| Ferritin only | 175(28.3) | 174(28.9) | 39(31) | 264(17.8) | 264(18) | 105(18.2) | 185(19.6) | 181(20) | 102(22) |
| TSAT only | 14(2.3) | 11(1.8) | 9(7.1) | 41(2.8) | 39(2.7) | 26(4.5) | 17(1.8) | 18(2) | 15(3.2) |
| Ferritin and TSAT | 104(16.8) | 96(15.9) | 46(36.5) | 426(28.6) | 410(28) | 267(46.3) | 139(14.7) | 119(13.2) | 153(33) |
| Other recommend laboratory testing, *n (%)* | | | | | | | | | |
| Liver enzymes (ALT or AST) | 467(75.4) | 452(75.1) | 92(73.0) | 1275(85.7) | 1254(85.7) | 526(91.2) | 780(82.7) | 750(83.0) | 391(84.3) |
| Serum/plasma creatinine | 614(99.2) | 598(99.3) | 124(98.4) | 1486(99.9) | 1463(99.9) | 577(100) | 943(100) | 904(100) | 464(100) |
| CRP | 458(74) | 444(73.8) | 106(84.1) | 1410(94.8) | 1389(94.9) | 562(97.4) | 921(97.7) | 881(97.5) | 450(97.0) |
| Other recommend procedures, *n (%)* | | | | | | | | | |
| Colonoscopy | 24(3.9) | 22(3.7) | 15(11.9) | 68(4.6) | 67(4.6) | 59(10.2) | 38(4.0) | 32(3.5) | 46(9.9) |
| Urinalysis | 15(2.4) | 16(2.7) | 9(7.1) | 31(2.1) | 31(2.1) | 22(3.8) | 15(1.6) | 13(1.4) | 13(2.8) |
| Cystoscopy | 37(6.0) | 35(5.8) | 6(4.8) | 66(4.4) | 66(4.5) | 29(5.0) | 61(6.5) | 61(6.7) | 39(8.4) |
| Esofagogastroduodenoscopy | 31(5.0) | 32(5.3) | 25(19.8) | 164(11.0) | 153(10.5) | 157(27.2) | 118(12.5) | 101(11.2) | 125(26.9) |
| Treatment patterns, *n (%)* | | | | | | | | | |
| Iron (oral or intravenous) | 148(23.9) | 139(23.1) | 67(53.2) | 483(32.5) | 482(32.9) | 212(36.7) | 212(22.5) | 207(22.9) | 131(28.2) |
| Blood transfusion | 21(3.4) | 19(3.2) | 10(7.9) | 75(5) | 67(4.6) | 91(15.8) | 88(9.3) | 74(8.2) | 108(23.3) |
| ESA | 0(0.0) | 0(0.0) | 0(0.0) | 0(0.0) | 0(0.0) | 3(0.5) | 2(0.2) | 2(0.2) | 4(0.9) |
| Combination treatment | 13(2.1) | 13(2.2) | 25(19.8) | 82(5.5) | 71(4.8) | 127(22) | 65(6.9) | 46(5.1) | 116(25) |
| Iron + blood transfusion | 10(1.6) | 10(1.7) | 19(15.1) | 78(5.2) | 67(4.6) | 107(18.5) | 62(6.6) | 43(4.8) | 106(22.8) |
| Iron + ESA | 2(0.3) | 2(0.3) | 3(2.4) | 0(0.0) | 0(0.0) | 11(1.9) | 3(0.3) | 3(0.3) | 7(1.5) |
| Blood transfusion + ESA | 1(0.2) | 1(0.2) | 2(1.6) | 2(0.1) | 2(0.1) | 3(0.5) | 0(0.0) | 0(0.0) | 3(0.6) |
| Iron + blood transfusion + ESA | 0(0.0) | 0(0.0) | 1(0.8) | 2(0.1) | 2(0.1) | 6(1) | 0(0.0) | 0(0.0) | 0(0.0) |
| Total population receiving treatment | 182(29.4) | 171(28.4) | 102(81.0) | 640 (43.0) | 620(42.3) | 433(75.0) | 367(38.9) | 329(36.4) | 359(77.4) |

**Abbreviations**: ALT, Alanine aminotransferase; AST, Aspartate aminotransferase; CRP, C-reactive protein; ESA, erythropoietin-stimulating agent; TSAT, transferrin saturation.

### **Table S9.** Clinical work-up of anemia after excluding patients died in the first 6 to 12 month after incident anemia.

|  | **Any anemia** | **Mild/moderate anemia** | **Severe anemia** |
| --- | --- | --- | --- |
| No. of anemia cases | 2770 | 2693 | 1054 |
| Received an anemia diagnosis, *n (%)* | 438 (15.8) | 392 (14.6) | 520 (49.3) |
| Testing of iron stores, *n (%)* | 1250 (45.1) | 1196 (44.4) | 696 (66.0) |
| Ferritin only | 574 (20.7) | 566 (21) | 224 (21.3) |
| TSAT only | 63 (2.3) | 60 (2.2) | 46 (4.4) |
| Ferritin and TSAT | 613 (22.1) | 570 (21.2) | 426 (40.4) |
| Other recommend laboratoty testing, *n (%)* |  |  |  |
| Liver enzymes (ALT or AST) | 2293 (82.8) | 2229 (82.8) | 916 (86.9) |
| Serum/plasma creatinine | 2765 (99.8) | 2689 (99.9) | 1052 (99.8) |
| C-reactive protein | 2520 (91) | 2447 (90.9) | 1008 (95.6) |
| Other recommend procedures, *n (%)* |  |  |  |
| Colonoscopy | 115 (4.2) | 107 (4.0) | 109 (10.3) |
| Urinalysis | 58 (2.1) | 57 (2.1) | 41 (3.9) |
| Cystoscopy | 146 (5.3) | 145 (5.4) | 66 (6.3) |
| Esophagogastroduodenoscopy | 272 (9.8) | 246 (9.1) | 283 (26.9) |
| Treatment patterns, *n (%)* |  |  |  |
| Iron, oral | 296 (10.7) | 283 (10.5) | 189 (17.9) |
| Iron, intravenous | 453 (16.4) | 451 (16.7) | 145 (13.8) |
| Iron, oral + intravenous | 32 (1.2) | 32 (1.2) | 46 (4.4) |
| Blood transfusion | 166 (6) | 143 (5.3) | 184 (17.5) |
| ESA | 2 (0.1) | 2 (0.1) | 7 (0.7) |
| Combination treatment | 144 (5.2) | 116 (4.3) | 245 (23.2) |
| Iron + blood transfusion | 135 (4.9) | 107 (4.0) | 212 (20.1) |
| Iron + ESA | 5 (0.2) | 5 (0.2) | 19 (1.8) |
| Blood transfusion + ESA | 2 (0.1) | 2 (0.1) | 7 (0.7) |
| Iron + blood transfusion + ESA | 2 (0.1) | 2 (0.1) | 7 (0.7) |
| Total population receiving any treatment | 1093 (39.5) | 1027 (38.1) | 816 (77.4) |

**Abbreviations**: ALT, Alanine aminotransferase; AST, Aspartate aminotransferase; ESA, erythropoietin-stimulating agent; TSAT, transferrin saturation.

### **Table S10.** Subgroup analyses: Adjusted hazard ratios of incident anemia and study outcomes

| **Subgroups** | **All-cause death** | | **MACE** | | **HF hospitalization** | |
| --- | --- | --- | --- | --- | --- | --- |
|  | **Adjusted HR (95% CI)** | **P-value for interaction** | **Adjusted HR (95% CI)** | **P-value for interaction** | **Adjusted HR (95% CI)** | **P-value for interaction** |
| **Type of HF** |  |  |  |  |  |  |
| HFrEF | 2.35 (1.98-2.79) | <0.001 | 2.50 (2.07-3.03) | 0.009 | 5.52 (4.69-6.50) | 0.235 |
| HFpEF | 1.85 (1.58-2.17) |  | 1.80 (1.47-2.21) |  | 4.28 (3.56-5.15) |  |
| **Sex** |  |  |  |  |  |  |
| Male | 2.26 (1.90-2.68) | 0.003 | 2.29 (1.88-2.79) | 0.089 | 4.99 (4.19-5.95) | 0.999 |
| Female | 1.89 (1.61-2.21) |  | 2.06 (1.69-2.51) |  | 4.81 (4.06-5.70) |  |
| **Diabetes status** |  |  |  |  |  |  |
| No | 2.01 (1.74-2.32) | 0.353 | 2.06 (1.74-2.45) | 0.883 | 4.53 (3.90-5.26) | 0.120 |
| Yes | 2.14 (1.75-2.62) |  | 2.34 (1.84-2.97) |  | 5.53 (4.48-6.82) |  |

**Abbreviations**: CI, confidence interval; HF, heart failure; HFpEF, heart failure with preserved ejection fraction; HFrEF, heart failure with reduced ejection fraction; HR, hazard ratio; MACE, major adverse cardiovascular event.

**Note**: MACE was defined as a composite of nonfatal stroke, nonfatal myocardial infarction, and cardiovascular death. The analyses for all adverse outcomes were adjusted for age, sex, education level, calendar year, baseline eGFR, baseline hemoglobin, diabetes, hypertension, ischemic heart disease, cerebrovascular disease (includes stroke), peripheral vascular disease, atrial fibrillation, valve disease, chronic obstructive pulmonary disease, rheumatoid diseases, dementia, liver disease, peptic ulcer disease, melanoma, device therapies, ACE inhibitors /angiotensin II receptor blockers (ARBs), beta-blockers, calcium channel blockers, loop diuretics, mineralocorticoid receptor antagonists, digoxin, statins, immunosuppressants, platelet inhibitors, anticoagulants, non-steroidal anti-inflammatory drugs, other blood pressure medications.

### **Table S11.** Comparison of traditional and competing risk models for study outcomes

| Outcomes | Traditional cox model | | Fine & Gray model | |
| --- | --- | --- | --- | --- |
|  | Crude hazard ratio (95% CI) | Adjusted hazard ratio (95% CI) | Crude subhazard ratio (95% CI) | Adjusted subhazard ratio (95% CI) |
| MACE | 2.75 (2.53-3.00) | 2.13 (1.85-2.44) | 2.13 (1.97-2.32) | 1.99 (1.74-2.27) |
| HF hospitalization | 4.78 (4.44-5.16) | 4.85 (4.30-5.48) | 2.81 (2.62-3.01) | 2.98 (2.64-3.35) |
| Cancer | 4.06 (3.71-4.45) | 3.41 (3.09-3.77) | 1.97 (1.81-2.14) | 1.91 (1.68-2.18) |

**Abbreviations**: CI, confidence interval; HF, heart failure; HR, hazard ratio; MACE, major adverse cardiovascular event.

**Note**: MACE was defined as a composite of nonfatal stroke, nonfatal myocardial infarction, and cardiovascular death. The analyses for all adverse outcomes were adjusted for age, sex, education level, calendar year, baseline estimated glomerular filtration rate, baseline hemoglobin, diabetes, hypertension, ischemic heart disease, cerebrovascular disease (includes stroke), peripheral vascular disease, atrial fibrillation, valve disease, chronic obstructive pulmonary disease, rheumatoid diseases, dementia, liver disease, peptic ulcer disease, melanoma, device therapies, ACE inhibitors /angiotensin II receptor blockers (ARBs), beta-blockers, calcium channel blockers, loop diuretics, mineralocorticoid receptor antagonists, digoxin, statins, immunosuppressants, platelet inhibitors, anticoagulants, non-steroidal anti-inflammatory drugs, other blood pressure medications.


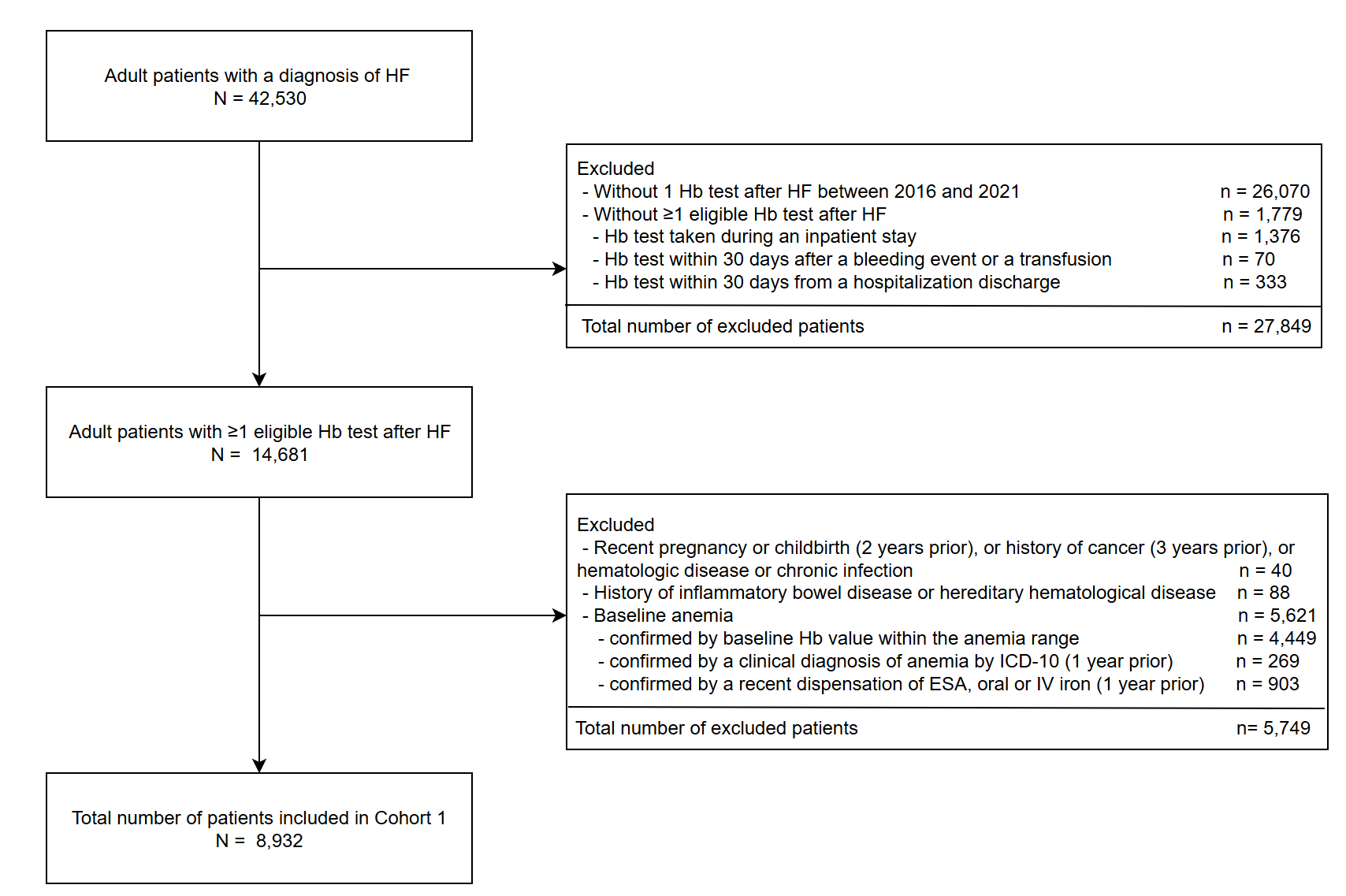


### **Figure S1.** Patient selection flowchart

**Abbreviations**: ESA, erythropoietin stimulating agent; Hb, hemoglobin; HF, heart failure; IV, intravenous.


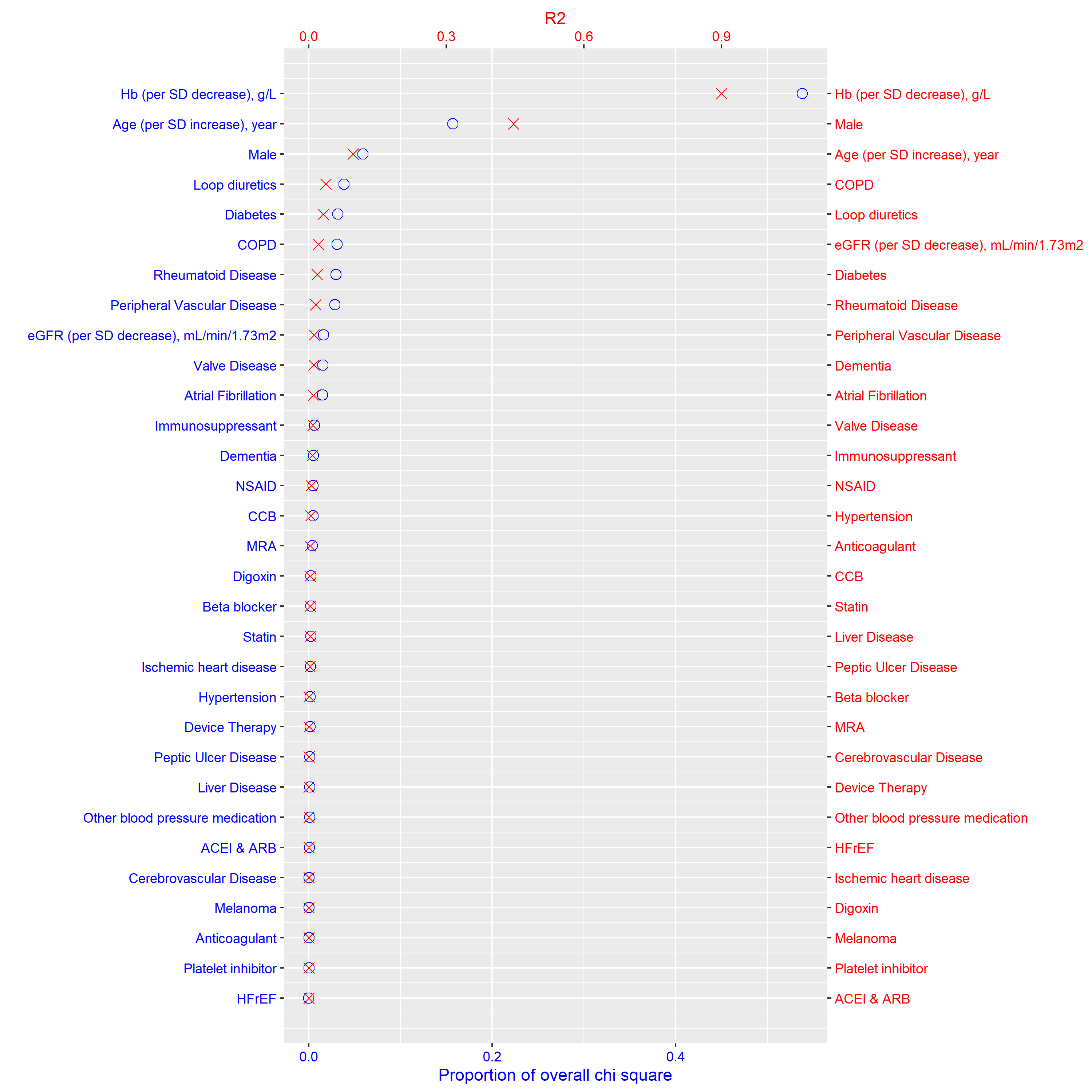


### **Figure S2.** Strength of the multivariable association between baseline conditions associated with incident anemia

**Abbreviations**: ACEI, angiotensin-converting enzyme inhibitor; ARB, angiotensin II receptor blocker; CCB, calcium channel blockers; COPD, chronic obstructive pulmonary disease; eGFR, estimated glomerular filtration rate; HFrEF, heart failure with reduced ejection fraction; MRA, mineralocorticoid receptor antagonists; NSAID, nonsteroidal anti-inflammatory drug.

**Note**: Strength of the multivariable association between baseline conditions associated with incident anemia was evaluated according to the estimated explained relative risk (R2, in red), and the proportion of overall explainable log-likelihood (Χ2, in blue). Hemoglobin (Hb) values were standardized as per standard deviation (SD) decrease.

**
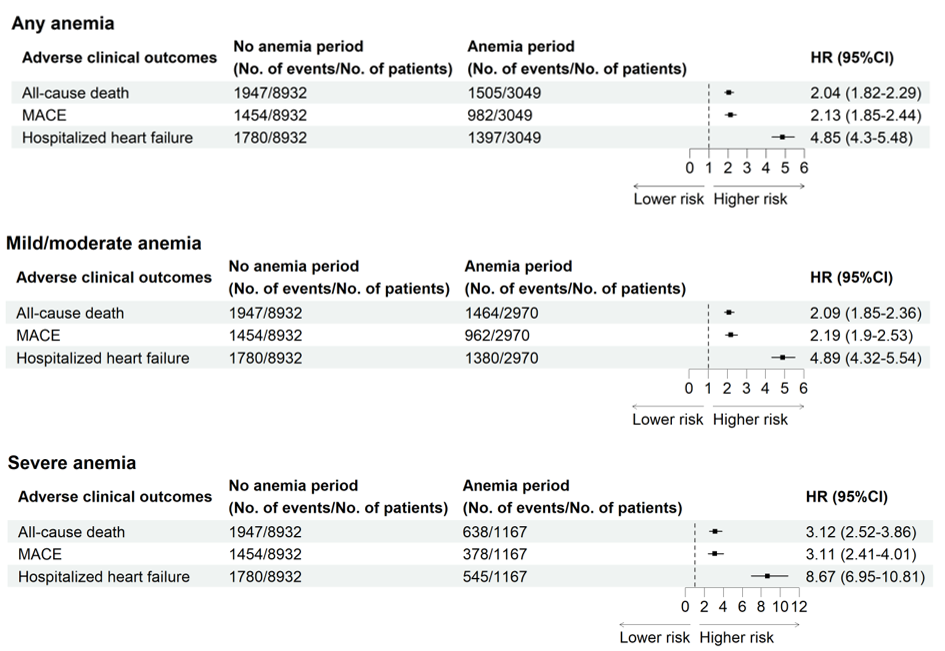
**

### **Figure S3.** Adverse clinical outcomes associated developing anemia in patients with heart failure

**Abbreviations**: CI, confidence interval; HR, hazard ratio; MACE, major adverse cardiovascular event.

**Note**: MACE was defined as a composite of nonfatal stroke, nonfatal myocardial infarction, nonfatal heart failure, and cardiovascular death. The analyses for all adverse outcomes were adjusted for age, sex, education level, calendar year, baseline estimated glomerular filtration rate, baseline hemoglobin, diabetes, hypertension, ischemic heart disease, cerebrovascular disease (includes stroke), peripheral vascular disease, atrial fibrillation, valve disease, chronic obstructive pulmonary disease, rheumatoid diseases, dementia, liver disease, peptic ulcer disease, melanoma, device therapies, ACE inhibitors /angiotensin II receptor blockers (ARBs), beta-blockers, calcium channel blockers, loop diuretics, mineralocorticoid receptor antagonists, digoxin, statins, immunosuppressants, platelet inhibitors, anticoagulants, non-steroidal anti-inflammatory drugs, other blood pressure medications.
